# Coarse-Grained Molecular Modeling of Pore Network Evolution in Particle- and Surfactant-Laden Emulsions

**DOI:** 10.1021/acs.jpcb.6c01516

**Published:** 2026-06-24

**Authors:** Yiqun Xu, Jonathan P. Singer, Ryan B. Sills

**Affiliations:** † Department of Materials Science and Engineering, 5970 Rutgers University, Piscataway, New Jersey 08854, United States; ‡ Department of Mechanical and Aerospace Engineering, Rutgers University, Piscataway, New Jersey 08854, United States

## Abstract

Macropore-infused nanocomposite emulsion thermosets (MINETs) are porous materials formed from emulsions containing high loadings of surfactants and nanoparticles, such as silica, activated carbon, alumina, and zinc oxide dispersed in the fluid mixture. After high-shear mixing, phase separation leads to the formation of a stable porous solid with a pore size that scales with the particle size. The mechanisms governing this pore network formation remain unclear. Here, we develop a coarse-grained molecular dynamics model to investigate phase separation and pore evolution in four-component systems composed of immiscible fluids, surfactants, and solid particles. Pair-correlation analysis is used to quantify domain coarsening, while pore size distributions are characterized using α-shape surface reconstruction. Results show that particles significantly alter phase separation kinetics, producing anomalous correlation-length scaling and suppressing pore coarsening. Pore size distributions evolve from monomodal to bimodal forms, with particles promoting finer pore structures. Increased surfactant concentration and near-neutral particle–fluid interactions further enhance pore stability. These findings provide mechanistic insight into MINET formation and guidance for designing porous nanocomposites with controlled microstructures.

## Graphical abstract

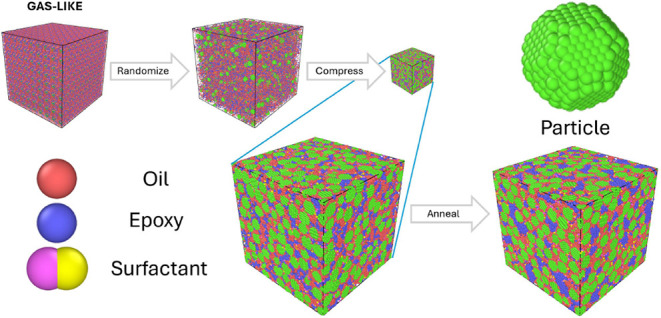

## Introduction

Porous materials with adjustable microstructures have garnered significant interest due to their wide-ranging applications in tissue engineering, catalysis, filtration, fuel cells, and sensors.^1^ Particle-laden emulsion systems, such as bicontinuous interfacially jammed emulsions (bijels),^2^ polymer high internal phase emulsions (polyHIPEs)^1,3^ capillary suspensions,^4^ and macropore-infused nanocomposite emulsion thermosets (MINETs),^5^ enable generation of porous materials with a wide range of microstructures. In these systems, pore networks result from phase separation of immiscible fluids (e.g., oil and epoxy, possibly containing surfactant) under the influence of particle-fluid and particle–particle interactions. The associated pore formation mechanisms depend on the relative volume fractions of the various phases and the wettability of the particles.^4^ For example, in bijels particles jam to stabilize the pore network^6^ whereas in capillary suspensions, capillary forces between particles are the main source of stability.^4^

In this work, we focus on the recently discovered MINET system,^5^ which differs from other emulsion-based techniques with a high particle volume fraction (up to 60%) and a surfactant volume fraction up to 10%. Previous research had found that such high particle volume fractions result in granular systems that do not form stable solids.^4^ However, MINETs form stable solids with a well-defined pore structure, enabling production of novel porous composites for a wide range of applications. A key feature of the MINET process is that it involves a single high-shear mixing step and remains processable in a manner similar to conventional epoxy resins, as shown in Figure 1. Preferential wetting of the particles to the epoxy leads to a particulate epoxy composite. The oil is then optionally rinsed away, depending on the applications of interest for the material. While experimental research has demonstrated the robustness and tunability of MINETs,^5,7,8^ the mechanisms underlying MINET formation and stability are poorly understood. This study investigates the mechanisms of MINET formation, focusing on pore network evolution.

**Figure 1:**
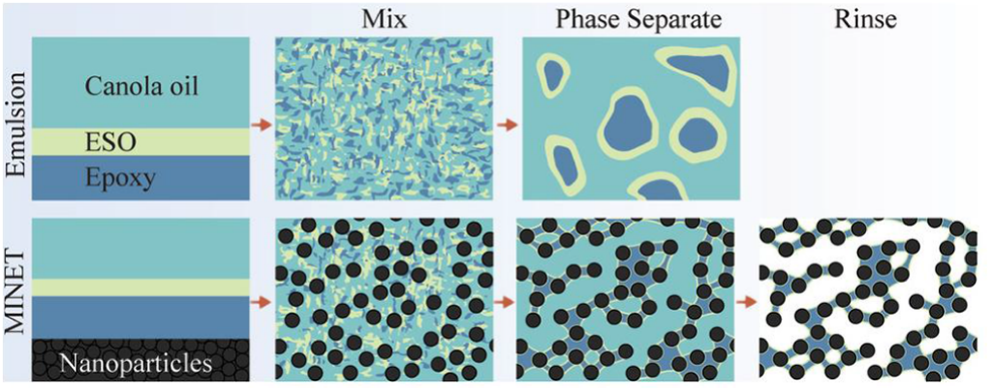
Comparison between traditional emulsions and macropore-infused nanocomposite emulsion thermosets (MINETs). In the conventional emulsion system (top row), the Canola oil–ESO­(surfactant)–Epoxy mixture forms dispersed domains during mixing and phase separation. In the MINET system (bottom row), the addition of nanoparticles arrests the evolving bicontinuous morphology, and after curing of the epoxy phase and removal of the oil phase, an interconnected porous structure remains. Reproduced from ref 5. Available under a CC BY-NC-ND 4.0 license. Copyright Hasan et al.

Several techniques exist for simulating phase-separating fluid systems, including phase field/lattice Boltzmann^9,10^ level set,^11^ and coarse-grained molecular dynamics (CG-MD) methods. While numerous studies have explored phase separating fluid systems containing surfactant and particles, to our knowledge no study has considered all four species (both fluids, surfactant, and particles) together. For these reasons, we focus here on developing and applying a simple CG-MD model to begin exploring the dynamics of pore network evolution in MINETs.

Numerous studies have significantly advanced our understanding of phase separation in multicomponent fluid systems using MD. Meyer et al. performed what is perhaps the first MD study of an immiscible liquid interface using a Lennard-Jones (LJ) potential, analyzing the density and pressure profiles near the interface.^12^ Smit et al. built on this work by including surfactant molecules in the simulation, observing the spontaneous formation of surfactant micelles.^13^ Subsequent works more thoroughly analyzed similar immiscible fluid interfaces^14,15^ with manually constructed interfaces. In contrast, Ma et al. started with a well-mixed binary fluid system and then evaluated the kinetics of phase separation by analyzing spatial correlations of the molecules (see next section for a detailed discussion on these calculations), which enables extraction of a characteristic length scale for the phase separation process.^16,17^ Ahmad et al.^18,19^ and Singh and Puri^20^ extended this analysis to binary and ternary fluid mixtures, respectively. Laradji et al. then incorporated the influence of surfactant in their analysis.^21^ Other works have further explored phase separation in confined regions.^17,22–24^ Li and Strachan considered the case of reaction-induced phase separation in a LJ-based epoxy-thermoplastic system.^25^ Going beyond simple LJ fluid systems, Ferrari et al.,^26^ Paul et al.,^27^ and Chen and Zheng^28^ studied phase separation in more complex water-based solutions.

Building on these studies, this research employs CG-MD simulations to examine the dynamics of MINET formation. After developing techniques to analyze pore size distribution in our simulations, we perform and analyze a large set of CG-MD simulations which span a range of fluid–fluid and fluid-particle interactions. Our results provide insights into the MINET formation process, which we hope will guide the development of MINET microstructures with tailored porosity and properties.

## Methods

### Coarse-Grained Molecular Dynamics Simulations

CG-MD is a computational modeling technique used to simulate the behavior of large molecular systems over longer length and time scales than all-atom MD. CG-MD simplifies the system by grouping multiple atoms into coarse-grained “beads”, representing larger units such as monomers, amino acids, nucleotides, or lipid molecules. Effective potentials describe the interactions between these coarse-grained beads, capturing essential system features.

In this first study on MINET formation, we utilize a simplified Lennard-Jones-based CG-MD model implemented in LAMMPS.^29^ The model contains two immiscible liquid species representing the oil and epoxy phases in MINETs, where oil serves as the porogen phase and epoxy represents the resin-forming phase. Although the experimental epoxy component is a larger thermosetting resin rather than a simple small molecule, here both liquid phases are coarse-grained into single-bead species in order to focus on the generic phase-separation and pore-evolution behavior prior to curing. These beads interact according to a Lennard-Jones potential with the form:

1 $$U_{ij}\left(r\right)=\{\begin{array}{ll} 4\varepsilon_{ij}\left[{\left(\frac{\sigma }{r}\right)}^{6}-{\left(\frac{\sigma }{r}\right)}^{12}\right], & r\leq r_{ij}^{c}\\ 0, & r>r_{ij}^{c} \end{array}$$

where *i* and *j* denote the species for the interacting pair of beads (*e* = epoxy, *o* = oil, *p* = particle), *ε*_*ij*_ is the interaction energy (minimum potential energy), σ is the characteristic length scale of the potential, and $r_{ij}^{c}$ is the cutoff radius. In the present coarse-grained model, these particles represent a generic solid particulate phase in MINETs rather than any one specific particle chemistry. LJ-based models have been extensively used to study phase-separating fluid systems,^12,13,16,18,19^ so our study can directly leverage these insights. To model surfactant molecules, we rigidly bond oil and epoxy beads with a bond distance of σ. This bonding produces hybrid molecules containing both oil-like and epoxy-like segments. These species are amphiphilic and preferentially localize at the oil–epoxy interface, thereby acting as surfactants. Particles are modeled as spherical rigid bodies with radius *R* containing beads arranged in a face-centered cubic lattice with a lattice constant of 1.584σ (yielding a nearest-neighbor distance of σ). Unless otherwise stated, all results used a particle size of *R* = 6σ. Rigid constraints for surfactant molecules and particles are enforced using the “fix rigid” method in LAMMPS. To achieve phase separation between the oil and epoxy, we set the cutoff distance between the liquid phases to $r_{eo}^{c}=1.122\sigma$ (i.e., at the minimum in the potential well) which results in a purely repulsive interaction. For all other interactions the cutoff distance is set to $r_{ee}^{c}=r_{oo}^{c}=r_{pp}^{c}=r_{ep}^{c}=r_{op}^{c}=2.5\sigma$ . To put epoxy and oil on an equal footing, the interaction energies *ε*_*ee*_ = *ε*_*oo*_ = *ε*_*eo*_ = *ε* are set equal to each other. Interaction energies *ε*_*ep*_ and *ε*_*op*_ (between fluid and particle beads) were varied to explore their influence on pore network formation, as discussed below. Finally, all fluid beads have equal mass *m*, while all beads in particles have a mass of *m*/1000, because particles are constructed from many beads, using the same bead mass would result in unrealistically large particle masses, causing them to remain nearly immobile at the simulation temperature. All results are presented in dimensionless units in terms of *ε*, σ, and *m*.

Our simulation procedure is depicted in Figure 2, whereby we emulate MINET manufacturing (see Figure 1) by initializing a random liquid mixture and simulating the phase separation process. To accomplish this, we start by initializing the system in a gaseous state (i.e., well separated molecules and particles) with all species arranged on a simple cubic lattice; this guarantees that no unstable configurations will be introduced in the initial structure which would cause the simulation to crash. We then anneal the system at constant temperature and volume (NVT ensemble) with a “high” temperature of *T* = 17.2 *ε*/*k*_B_, allowing the species to fully mix (and erase any memory of the initial simple cubic arrangement). Subsequently, we reduce the temperature to *T* = 0.862*ε*/*k*_B_ and rapidly compress the system to condense it into a liquid by applying a hydrostatic strain rate of $-0.02\sqrt{\varepsilon /\left(m\sigma^{2}\right)}$ . We continue compressing the system until the void volume fraction is below 3%, thereby attaining a “fully condensed” liquid system. We use a probing sphere diameter of 2σ to compute the void volume fraction; see the next section for details on how this void volume fraction was determined. At the end of the compression step the number density is typically on the order of ∼0.26σ^–3^, which is in the liquid region of the single-species Lennard-Jones phase diagram.^30^ Finally, the system is again annealed at constant temperature and volume with a temperature of *T* = 0.862*ε*/*k*_B_ to allow phase separation to proceed. This temperature is well below the critical temperature of the single-species Lennard-Jones fluid ( $T_{c}^{*}\approx 1.32\varepsilon /k_{B}$ ,^31,32^ whose critical density is $\rho_{c}^{*}\approx 0.316\sigma^{-3}$ . In a pure-liquid reference system at this temperature, we obtain a density of ρ = 0.574σ^–3^, which is well within the condensed-liquid regime. The binary oil–epoxy system has a lower average density, ρ = 0.261σ^–3^.^31,32^ Although this value is lower than the single-component critical density, this comparison is only qualitative because the present system is a binary mixture rather than a single-component fluid. In addition, the cluster analysis of the binary system was performed in OVITO^33^ using a connectivity cutoff of 2.5σ, and more than ∼99.7% of atoms were found to belong to the largest connected cluster. Thus, the simulated structures are clearly dominated by liquid–liquid phase separation rather than by a gas-dominated state.

**Figure 2:**
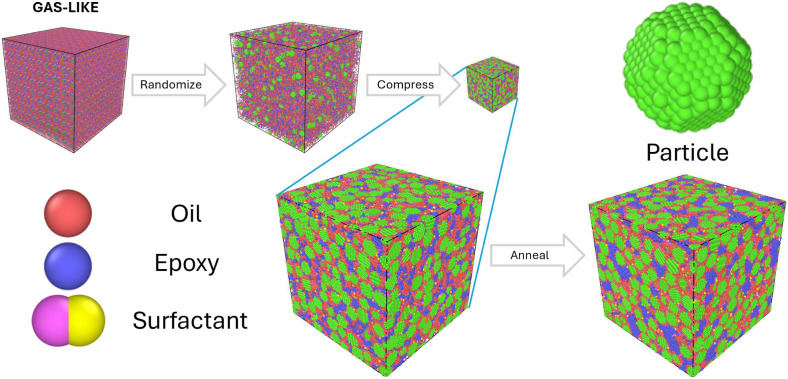
Schematic of the simulation procedure and model components. The system is initialized in a low-density simple-cubic arrangement, randomized at high temperature to generate a gas-like mixed state, and then compressed into a condensed liquid configuration. The compressed system is subsequently annealed at lower temperature to simulate phase separation and pore network evolution. Colors indicate oil (red), epoxy (blue), and surfactant (yellow/magenta), and the green cluster illustrates the coarse-grained particle structure used in the simulations.

We performed simulations with a range of particle sizes and surfactant concentrations. To keep our simulation conditions relevant to MINETs where the particle volume fraction is about 50%, we set the ratio of the number of fluid beads (sum of oil, epoxy, and surfactant beads) to the number of particle beads to 0.82, which we empirically determined yields particle volume fractions close to 50% after condensation. Furthermore, in the prior experimental study on MINETs, the ratio of oil and the sum of epoxy and surfactant was held constant. To emulate this stoichiometry, we set the ratio (*N*_*e*_ + *N*_*s*_)/*N*_*o*_ = 0.5, where *N*_*e*_ is the number of epoxy beads, *N*_*s*_ is the number of surfactant molecules (each containing two beads), and *N*_*o*_ is the number of oil beads. Finally, the surfactant concentration varies in our study and is specified in terms of the surfactant concentration, *c*_*s*_ = *N*_*s*_/(*N*_*e*_ + *N*_*o*_). The typical total number of fluid beads in our simulations was about 400,000, with the total number of beads about 1,100,000.

### Computation of Pore Size Distribution

Pore size distribution (PSD) refers to the range and frequency of pore sizes in a material. For microporous materials, understanding the PSD is crucial for assessing properties and applications since it influences adsorption capacity, filtration, flow characteristics, catalytic activity, and overall material structure. Experimentally, techniques such as mercury intrusion porosimetry (MIP) are used to quantify the pore size distribution.^34^ MIP assumes that all pores are cylindrical and uses a correlation between pressure and degree of fluid penetration to estimate the total volume of a given pore size. This procedure is impractical for our simulations because it would require a mercury-like coarse-grained probe molecule.

Thus, we developed a new method to measure PSD using the α-shape surface construction method in OVITO.^33,35^ The α-shape method represents the shape of a set of points in space using a “probing sphere” of user-defined diameter *d*_*probe*_. Conceptually, the method works by probing the collection of points (which correspond to the molecules in our simulations) using the sphere by translating it throughout the entire simulation volume. Everywhere that the sphere is not penetrated by any points and comes in contact with three points, a triangular surface mesh face is inserted. The net result is a surface mesh that defines the geometry of the point cloud for a given probing sphere radius, as shown in Figure 3. Volumes and surface areas for the underlying pore representation can be easily computed. If the probing sphere is smaller than the minimum separation distance between the points, no surface mesh is produced. Similarly, if the probing sphere is too large, there is no void space large enough to fit the sphere (without being penetrated by one or more points) and no surface mesh is produced.

**Figure 3:**
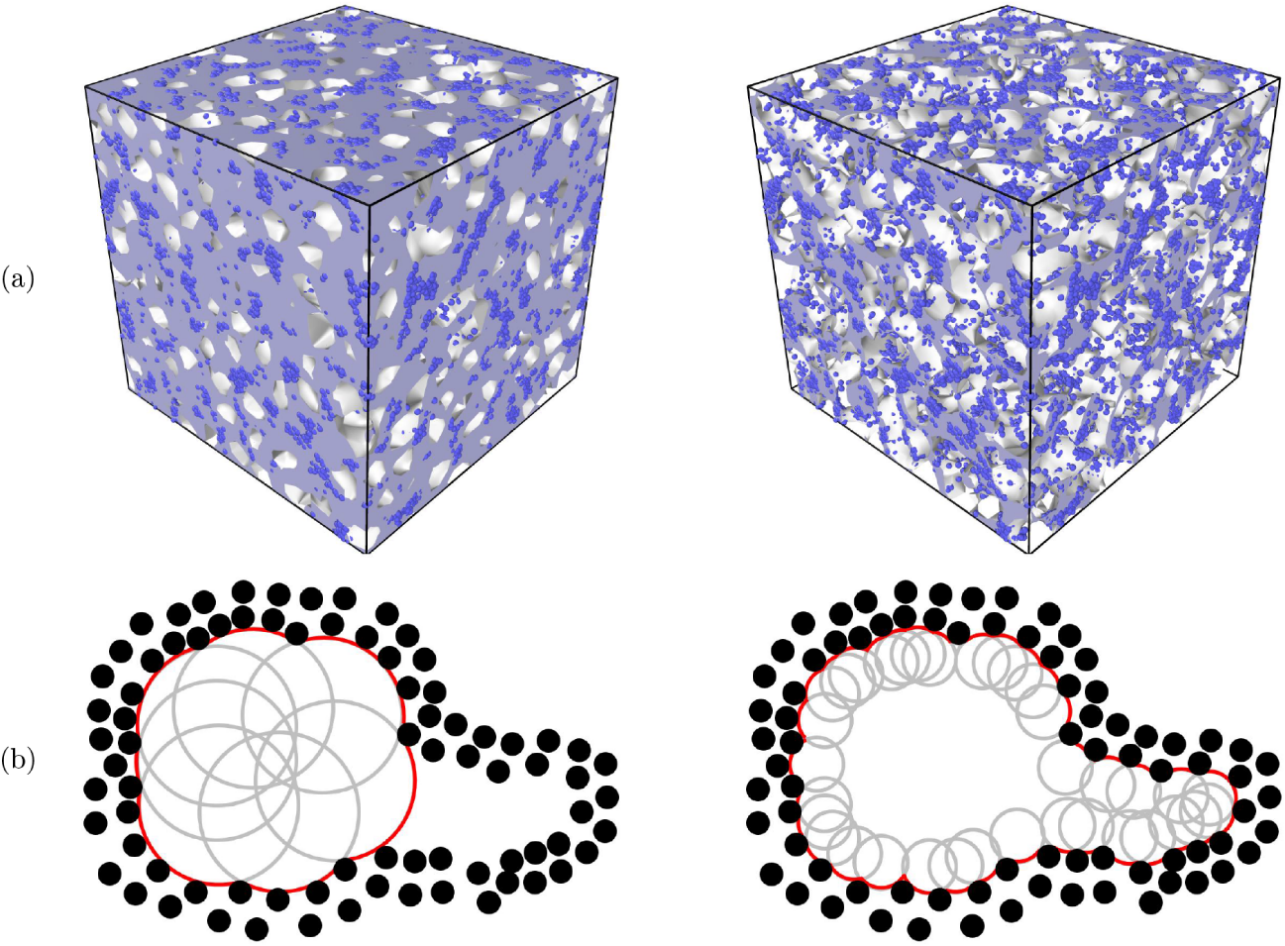
Procedure used to calculate pore size distributions (PSDs). (a) Examples of pore-space surface meshes generated in OVITO with the alpha-shape method using different probing-sphere radii; decreasing the probe radius allows smaller pore features to be resolved. (b) Schematic illustrating this effect: a larger probing sphere cannot enter narrow constrictions, whereas a smaller probing sphere can access smaller pores and more confined regions of the pore network.

Here, we exploit the fact that the probing sphere diameter is user-defined to systematically probe smaller and smaller pores, thereby obtaining a PSD. To analyze the PSD, we first delete the oil molecules to simulate rinsing of oil in MINETs, leaving behind a porous region. As shown in Figure 3, using a larger probing sphere diameter *d*_1_ to construct the internal pore surface results in a smaller pore volume, *V*(*d*_1_), because the sphere cannot penetrate smaller pores. Conversely, a smaller probing sphere diameter *d*_2_ penetrates smaller pores, resulting in a larger pore volume *V*(*d*_2_). The difference between these volumes *V*(*d*_2_) – *V*(*d*_1_) corresponds to the pore volume with an average pore diameter of (*d*_1_ + *d*_2_)/2. By applying a series of *n* probing sphere diameters with increment Δ*d*, we can obtain a pore size probability density function,

2 $$\frac{dV\left(d=i\Delta d\right)}{d\log_{10}d}\approx \frac{V\left(d_{i}^{-}\right)-V\left(d_{i}^{+}\right)}{\log_{10}\left(d_{i}^{-}\right)-\log_{10}\left(d_{i}^{+}\right)}$$

where $d_{i}^{-}=i\Delta d-\Delta d/2$ and $d_{i}^{+}=i\Delta d+\Delta d/2$ are the lower and upper bounds of the pore-diameter bin centered at *d*_*i*_ = *i*Δ*d*, and *i* = 1,···,*n*. Thus, $V\left(d_{i}^{-}\right)-V\left(d_{i}^{+}\right)$ represents the pore volume associated with that size interval, and eq 2 is a finite-difference approximation of d*V*/dlog_10_ *d*. This definition is consistent with the PSDs produced via MIP.^34^ Similarly, we can compute the volume-weighted average pore size as

3 $$\left\langle d\right\rangle \approx \frac{\underset{i}{\sum }i\Delta d\Delta V\left(i\right)}{\underset{i}{\sum }\Delta V\left(i\right)},,\Delta V\left(i\right)=V\left(d_{i}^{-}\right)-V\left(d_{i}^{+}\right)$$

### Pair Correlation Analysis

Pair correlation analysis has been used extensively to evaluate phase separation in fluid systems.^18,25,26,36^ Here we perform a similar analysis to benchmark the behavior of our simulations and demonstrate the influence of surfactant and particles on phase separation. For this analysis, the molecular system is first coarse-grained through voxelization using a voxel width of *w*. Specifically, we use *w* = 2σ, which is only slightly larger than the bead diameter, thereby reducing local counting noise while preserving spatial resolution. At the same time, this voxel size remains much smaller than the particle radius (*R* = 6σ in the reference case), ensuring sufficient resolution of the phase-separated morphology without excessively smoothing particle-scale features. For each simulation time *t*, the order parameter ϕ for each voxel at position **x** is then computed as

4 $$\phi \left(x,t\right)=\frac{n_{e}\left(x,t\right)-n_{o}\left(x,t\right)}{n_{e}\left(x,t\right)+n_{o}\left(x,t\right)}$$

where *n*_*e*_(**x**,*t*) and *n*_*o*_(**x**,*t*) are the numbers of epoxy and oil beads, respectively, within the voxel. This order parameter varies between 1 when the voxel is filled with epoxy and −1 when filled with oil. When computing ϕ, we ignore surfactant molecules (since they contribute neutrally, with an epoxy head and an oil tail). Additionally, particles are treated as being filled with epoxy since they are preferentially wetted to the epoxy.

Next the pair correlation function *C*(*r*,*t*) describing the spatial variation of the order parameter is computed as

5 $$C\left(r,t\right)=\left\langle \phi \left(x_{1},t\right)\phi \left(x_{2},t\right)\right\rangle -\left\langle \phi \left(x_{1},t\right)\right\rangle \left\langle \phi \left(x_{2},t\right)\right\rangle$$

where *r* = ||*x*_1_ – *x*_2_|| is the separation distance between a pair of voxels and ⟨·⟩ denotes an average among all pairs of voxels with separation distance *r*. *C*(*r*,*t*) indicates on average how correlated or anticorrelated the ϕ values are between two points with separation distance *r*. When *C*(*r*,*t*) = 1, voxel pairs are perfectly correlated, meaning that ϕ always takes the same value at separation distance *r*. On the other hand, *C*(*r*,*t*) = −1 means that voxel pairs are perfectly anticorrelated (i.e., ϕ = 1 in one voxel and ϕ = −1 in the other). *C*(*r*,*t*) = 0 indicates no correlation. *C*(*r*,*t*) can be efficiently computed in Fourier space using a fast Fourier transformation.^18^

## Results

### Evolution of Phase Separation and Pore Size Distributions

For the simulations in this section, we take *ε*_*ep*_/*ε*_*ee*_ = 0.5 and *ε*_*ep*_/*ε*_*op*_ = 2, meaning that epoxy is attracted to itself more strongly than it is attracted to the particles and that particles favor epoxy over oil by a factor of 2 (in terms of the interaction energy). Figure 4 shows simulation snapshots over time for cases with (a) only the binary liquid, (b) the binary liquid with surfactant concentration *c*_*s*_ = 0.034, (c) binary liquid with particles of radius *R* = 6σ (no surfactant), and (d) the binary liquid with surfactant and particles. The different nondimensional times $t^{*}=t/\sqrt{\sigma^{2}m/\epsilon }$ are indicated in the figure. Phase separation over time is apparent in all cases. Comparing (a) and (b), surfactant is shown to retard the phase separation process. With the addition of particles in (c), the (red) oil domains are smaller at later times in comparison to both (a) and (b), indicating that particles are retarding phase separation even more effectively than surfactant. Finally, both (c) and (d) are similar, indicating a small influence of surfactant when particles are present.

**Figure 4:**
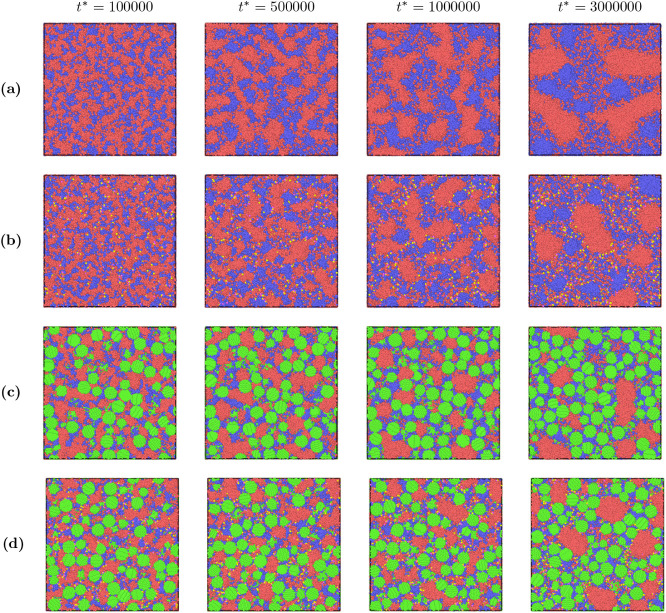
Snapshots from simulations showing phase separation behavior over time with (a) only the binary liquid, (b) binary liquid with surfactant, (c) binary liquid with particles, and (d) binary liquid with surfactant and particles. Showing results at nondimensional times of t* = 100,000, 500,000, 1,000,000, and 3,000,000.

Figure 5 presents correlation curves at the four different points in time shown in Figure 4a–d. The curves show similar behavior to prior works^18,25^ at small distances the system is highly correlated with *C* ≈ 1 which decays as the separation distance increases. At some distance, the correlation completely disappears when *C* ≈ 0, after which the system becomes slightly anticorrelated with *C* < 0. Finally, at large distances the system becomes uncorrelated (*C* ≈ 0).

**Figure 5:**
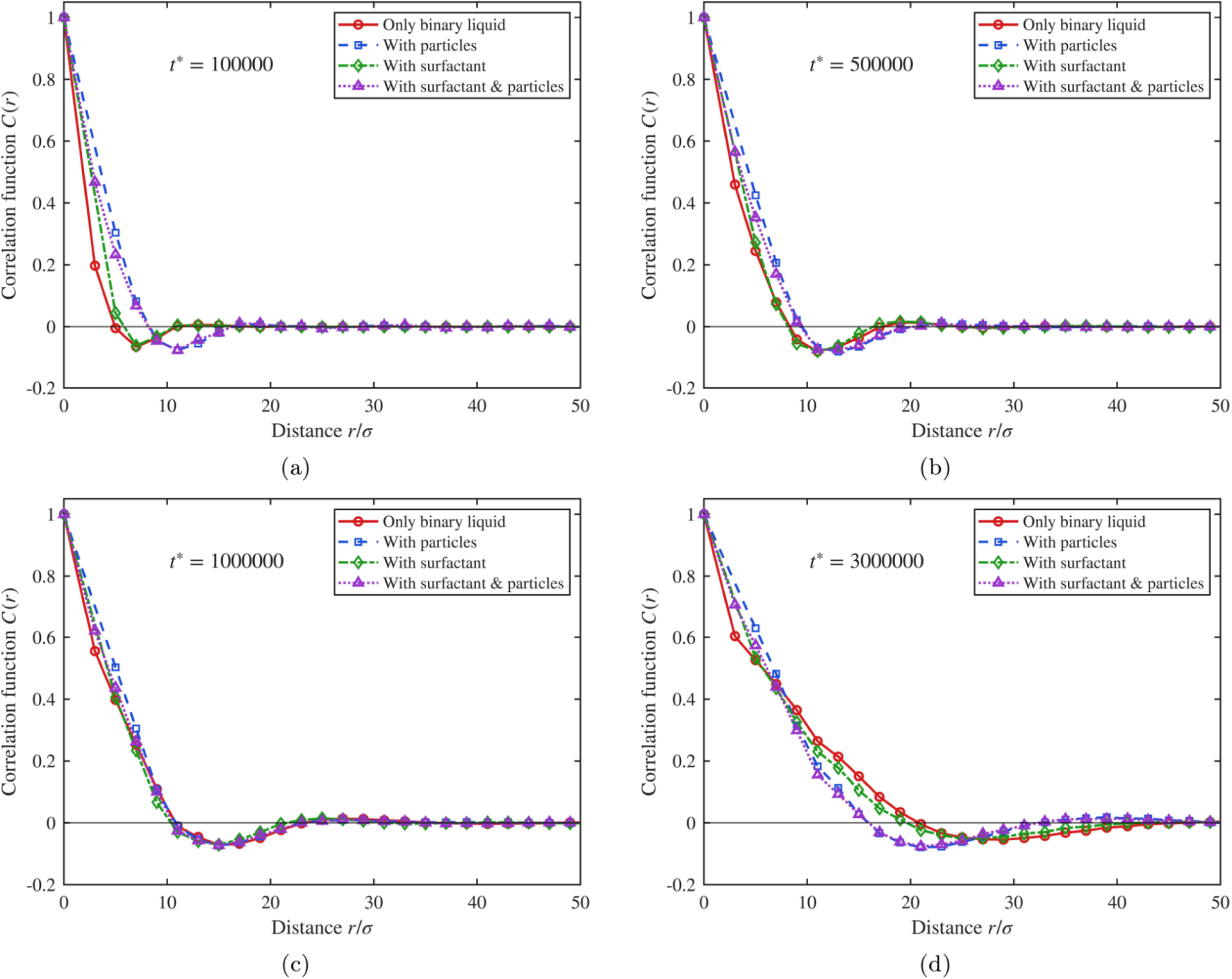
Correlation functions obtained from simulation results in Figure 4; (a)–(d) correspond to increasing time.

Comparing across the cases reveals the influence of surfactant and particles on phase separation. When t* = 100,000 the systems containing particles have phase separated more significantly than those without (correlations extend to larger length scales). However, when t* = 1,000,000 the particle-free systems have “caught up” to the particle-laden systems, since all four curves are essentially on top of each other. From there, the particle-free systems continue to phase separate more rapidly. The particle-free system containing surfactant phase separates slightly more slowly than the binary liquid, as expected, but this effect is small compared to the influence of the particles.

To further examine dynamic scaling, we replotted the correlation functions as a function of the rescaled distance $r/l\left(t^{*}\right)$ , where $l\left(t^{*}\right)$ is defined as the point where the curve first crosses *C* = 0 ^18^ and is used here as a measure of domain length. If the structures are self-similar over time, the curves at different times should collapse onto a single master curve. As shown in Figure 6, all four systems exhibit reasonably good collapse after rescaling by $l\left(t^{*}\right)$ , including the particle-containing cases. This indicates that, within the resolution of the present simulations, the evolving structures remain approximately self-similar in time and do not show clear evidence of fractal-like deviations from standard dynamic scaling.^37^

**Figure 6:**
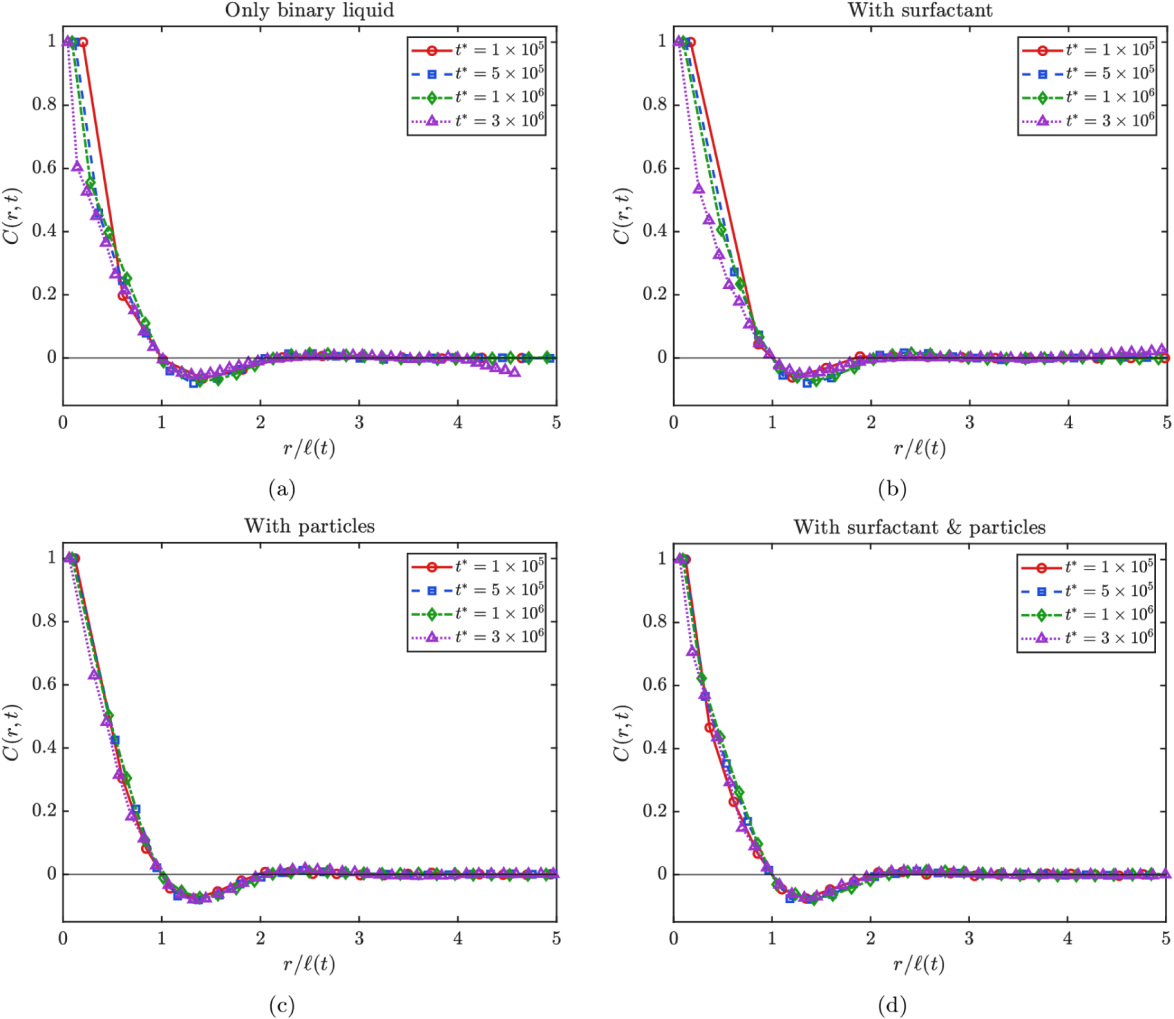
Correlation functions replotted against the rescaled distance $r/l\left(t^{*}\right)$ for the simulation results in Figure 4. Panels (a)–(d) correspond to the binary liquid only, with surfactant, with particles, and with surfactant and particles, respectively. The collapse of the curves at different times indicates approximate dynamic scaling.

This behavior becomes even clearer when the domain length l is analyzed, confirming that particle-free systems coarsen more rapidly. Figure 7a presents l as a function of time in a log–log plot. In three-dimensional phase separation, the expected sequence of growth regimes is diffusive, viscous hydrodynamic, and inertial hydrodynamic, with exponents α = 1/3, 1, and 2/3, respectively.^38–41^ We therefore include reference slopes corresponding to these exponents, along with a 1/6 guide to highlight the anomalously slow early time growth observed in the particle-containing cases. Because crossover behavior is difficult to identify reliably from the log–log plot alone, we also analyze the time-dependent effective exponent following prior work.^42,43^ Specifically, we define

6 $$n_{\mathrm{eff}}\left(t\right)=\frac{\log_{10}\left[l\left(5t^{*}\right)/l\left(t^{*}\right)\right]}{\log_{10}5}$$

which reduces to the true growth exponent when $l\left(t^{*}\right)\propto t^{\alpha }$ . Figure 7b shows *n*_eff_ plotted as a function of $1/l\left(t^{*}\right)$ , which provides a convenient way to examine the asymptotic growth behavior.

**Figure 7:**
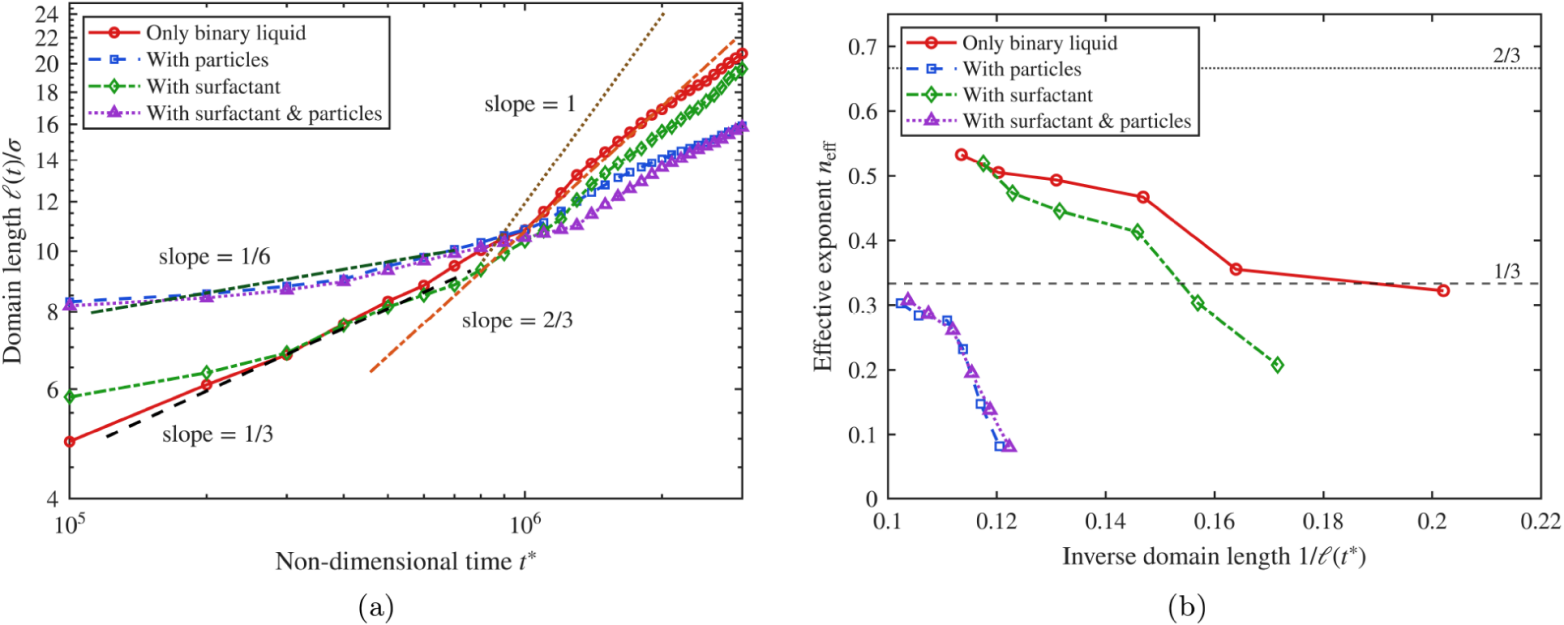
(a) Domain length $l\left(t^{*}\right)$ as a function of nondimensional time, extracted from the first zero crossing of the correlation functions in Figure 5. Reference slopes corresponding to α = 1/3, 1, 2/3, and 1/6 are shown for comparison. (b) Effective growth exponent *n*_eff_, defined by eq 6, plotted as a function of $1/l\left(t^{*}\right)$ .

In the particle-free cases, the early time growth is consistent with *n*_eff_ ≈ 1/3, as expected for diffusive coarsening. However, we do not observe the full expected sequence 1/3 → 1 → 2/3 within the accessible simulation window. Instead, the growth departs from 1/3 at later times without showing a clearly resolved viscous hydrodynamic regime with exponent 1 or an inertial regime with exponent 2/3, likely because the accessible times and system sizes are not sufficient to capture the full set of crossovers. In contrast, the particle-containing cases exhibit substantially smaller effective exponents, close to 1/6 at early times, indicating that the coarsening kinetics are fundamentally altered when particles are present. At later times, these particle-containing cases evolve toward larger exponents, but their behavior remains clearly distinct from that of the particle-free systems. Surfactants have a relatively negligible effect at this concentration; we will explore higher surfactant concentrations in later sections.

Next, we analyze the evolution of the pore sizes in the systems; correlation analysis reveals important insights into the nature of the phase separation but is not the most relevant feature of the system for porous materials. For this reason, we focus the rest of the work on analyzing the pores directly via the PSD.

Figure 8 presents PSD curves that mirror the plots presented in Figure 5 for cases with a binary liquid alone, with surfactant, with particles, and with surfactant and particles. At the beginning of the annealing step (a), the cases without particles exhibit a relatively narrower pore size distribution in comparison to the cases with particles. As time evolves (b), all cases develop a bimodal pore size distribution and the peak at larger pore sizes shifts to the right while the small pore peak remains relatively fixed in place (c,d). Both peaks for cases without particles are offset slightly to the right (e.g., larger pores) from the peaks with particles, and cases with particles feature a larger fraction of pores at very small and intermediate (between the peaks) sizes.

**Figure 8:**
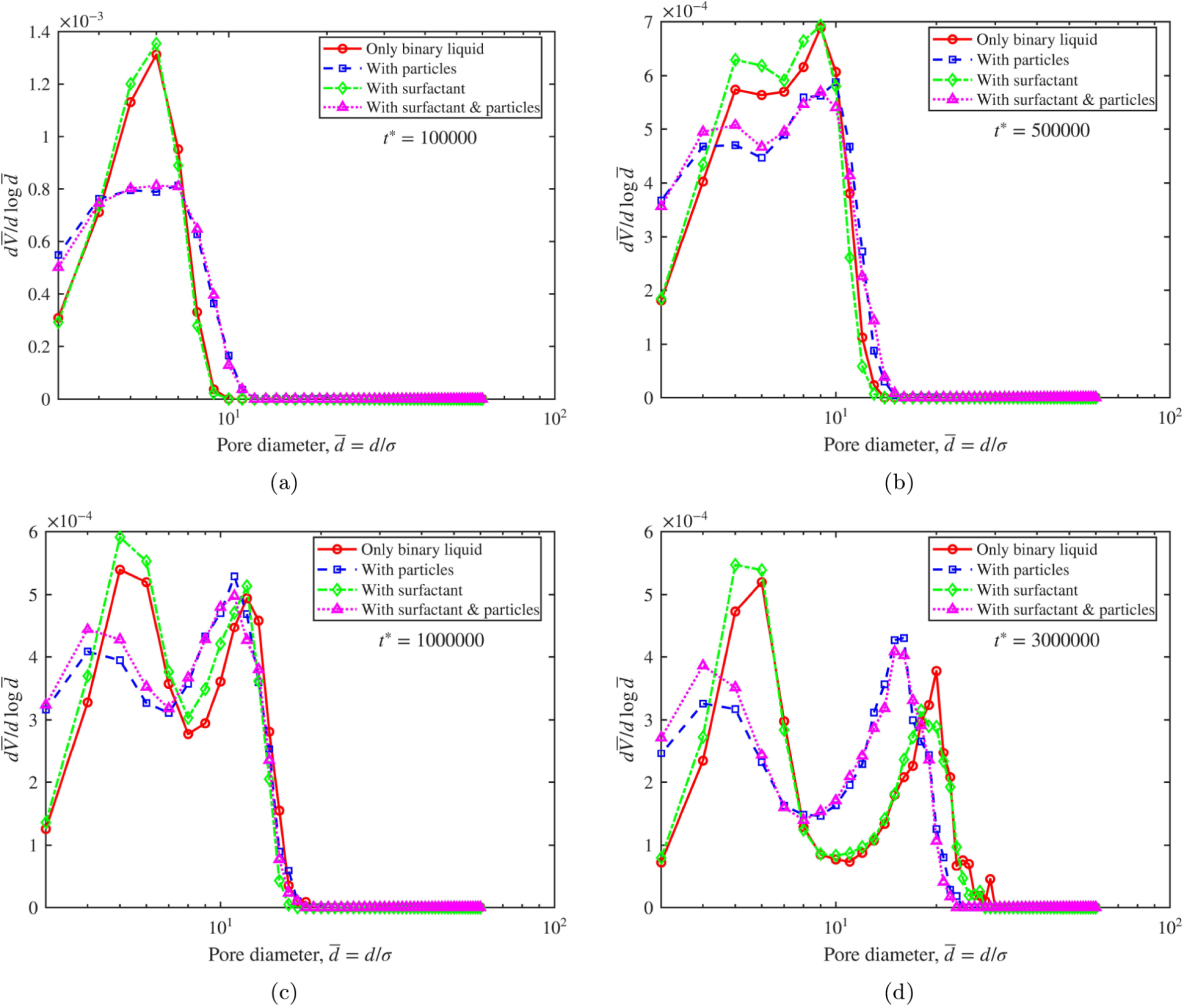
Pore size distributions for the simulation results from Figure 4; (a)–(d) correspond to increasing time.

We may also ask whether there are differences in the morphology of the epoxy network itself, rather than only in the pore structure obtained after removing the oil phase. In the primary PSD analysis above, we remove the oil and surfactants to mimic the experimental rinsing step and characterize the resulting pore network. However, because the same geometric procedure can also be applied to the complementary phase in the simulations, we can additionally remove the epoxy, particles, and surfactant and analyze the remaining oil/void phase. The resulting distributions therefore do not represent the experimentally retained pore phase, but instead provide an indirect measure of the epoxy morphology through its complementary geometry. Figure 9 shows these results. Particles are shown to have a huge effect on the epoxy morphology. Without particles, a smooth monomodal distribution is observed which transitions to a bimodal distribution over time. Surfactant acts to promote small pores. In contrast, when particles are present, the PSD has a single sharp peak at a pore size of about 10σ with no pores larger than this. This indicates that particles tend to yield epoxy morphologies with a well-defined length scale and minimal features away from that length scale.

**Figure 9:**
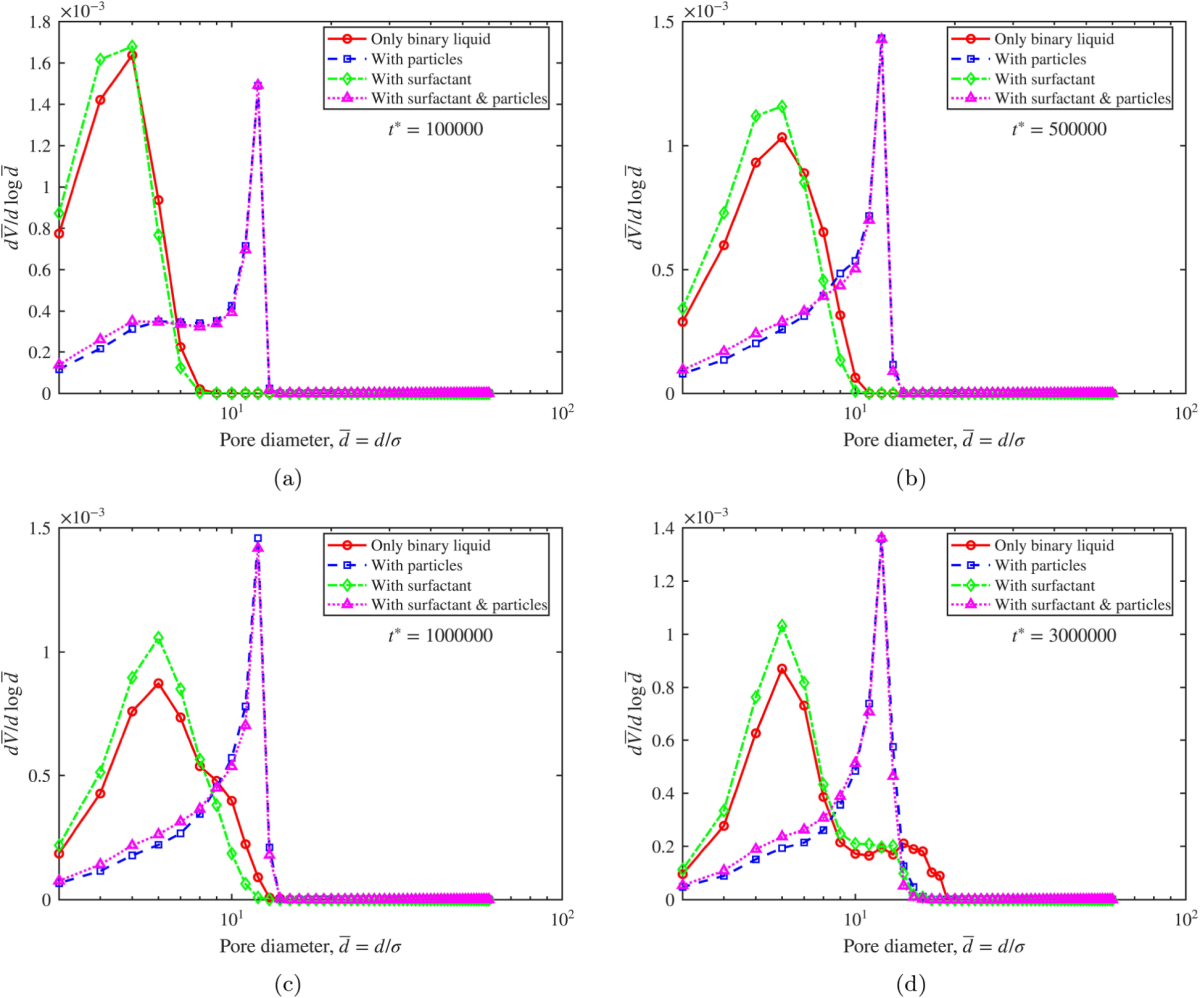
Pore size distributions computed for the complementary void (oil) phase of the simulation results in Figure 4, used here to characterize the morphology of the epoxy network. Panels (a)–(d) correspond to increasing nondimensional times t* = 100,000, 500,000, 1,000,000, and 3,000,000.

To reveal the overall trends in the PSD evolution over time more clearly, we plot the average pore diameter ⟨*d*⟩ as a function of time in Figure 10. This shows how the mean pore size systematically increases with time in all cases as phase separation progresses. We attempted to analyze these curves in terms of power law evolution, but found this description to be inconsistent with the time scaling of the mean pore size. Instead, after an initial transient phase, the mean pore size exhibits linear increase over time (as shown by the dashed lines), indicating a steady-state pore growth phase; we term this steady-state growth rate as ⟨*ḋ*⟩. The steady-state growth rate is shown to systematically decrease upon introduction of surfactant and particles, and is minimized when both are present in the emulsion. From the standpoint of MINETs, the slope of this curve indicates the stability of the pore structure since the epoxy must cure before the pore network disassembles itself with phase separation. The steady-state slope ⟨*ḋ*⟩ is a direct measure of the network stability. Hence, going forward, we consider how the steady-state slope varies with the features of the system.

**Figure 10:**
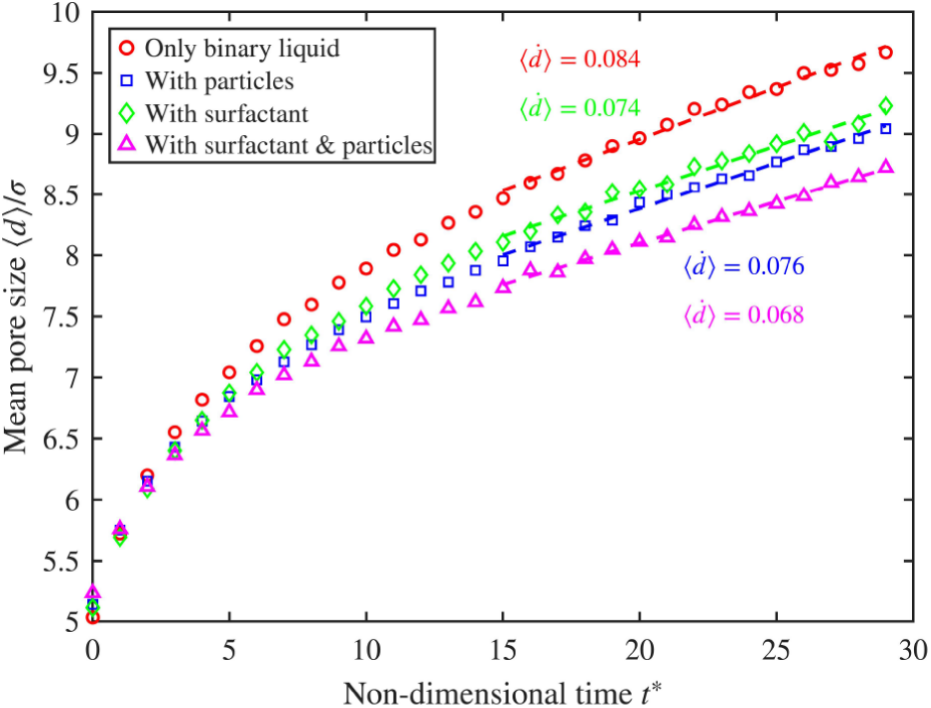
Average pore size over time for the PSDs in Figure 8. Steady-state pore growth rate ⟨*ḋ*⟩ is obtained from the dashed line fits.

### Influence of Composition and Interaction Energies

Next, we explore the influence of the MINET composition and interaction energies on the pore evolution. We take our previous configuration as the “baseline” and then explore variations beyond this configuration.

First, we consider the influence of particle size. Given the limitations of MD, we were only able to perform MD simulations with particle sizes of *R* = 6σ, 8σ, and 10σ. The resulting PSDs over time are shown in Figure 11a–c. As the particle size increases, the main effect is that the peak at larger particle sizes shrinks. In other words, the PSD becomes less bimodal as the particle size increases, consistent with experimental observations that a bimodal PSD is only observed with nanoscale particles, larger particles yield monomodal PSDs.^5^ On the other hand, our results do not show a strong connection between particle size and mean pore size, as shown in Figure 11d. This contradicts experimental evidence showing a correlation between particle size and pore size, albeit when comparing particle sizes that differ over a span of more than 2 orders of magnitude^5^ (compared to less than a factor of 2 here).

**Figure 11:**
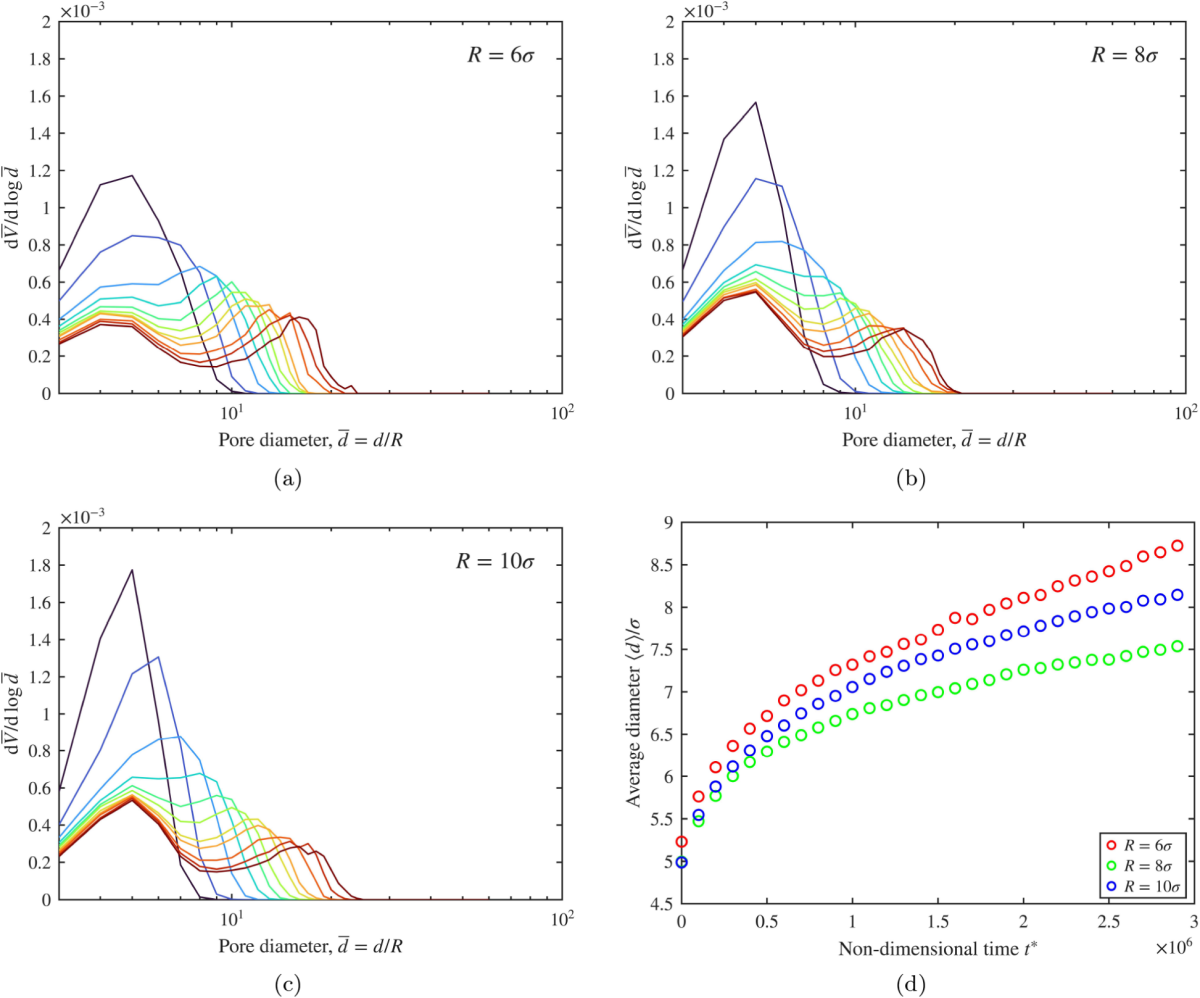
(a–c) Pore size distribution evolution over time with particle sizes *R*/σ = 6, 8, 10. In each panel, the different colored curves (dark → blue → green → red) correspond to increasing simulation time. (d) Mean pore size over time for results in (a–c).

Next, we explore the influence of surfactant concentration on the steady-state pore growth rate with and without particles, with results shown in Figure 12. In both cases, surfactant reduces the driving force for coarsening, while particles consistently further suppress growth. The influence of surfactant on the pore stability, as measured by the slope $\frac{d\left\langle ḋ\right\rangle }{dc}$ , is roughly the same whether particles are present or not. A surfactant concentration of *c*_*s*_ = 0.13 leads to a decrease of ⟨*ḋ*⟩ by about 30%, indicating significant stabilization of the pore network.

**Figure 12:**
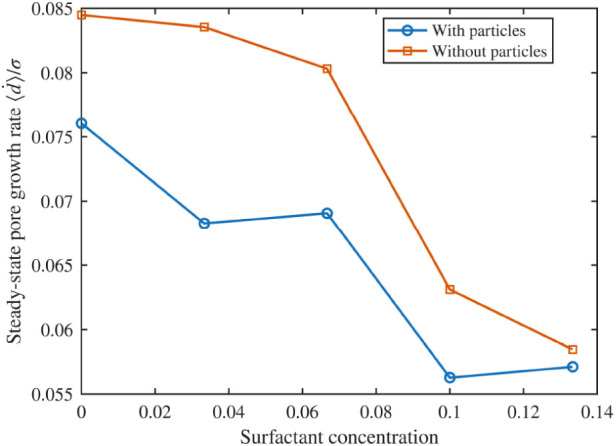
Steady-state growth rate as a function of surfactant concentration with and without particles when *ε_ep_*/*ε_ee_* = 0.5 and *ε_ep_*/*ε_op_* = 2.

Figure 13 examines the role of the various interaction energies in the model through a three-dimensional representation of the steady-state pore growth rate as a function of the ratios *ε*_*ep*_/*ε*_*ee*_ and *ε*_*ep*_/*ε*_*op*_; recall that *ε*_*ep*_/*ε*_*ee*_ specifies how strongly the epoxy interacts with particles in comparison to itself and *ε*_*ep*_/*ε*_*op*_ specifies the strength of the particle bias for epoxy relative to oil. As shown in the surface plot, increasing *ε*_*ep*_/*ε*_*op*_ correlates with faster pore evolution, indicating that when particles attract epoxy much more strongly than oil, structural rearrangements accelerate. This effect saturates, however, indicating that once the epoxy-oil asymmetry is large enough the magnitude of the asymmetry does not matter. Conversely, increasing *ε*_*ep*_/*ε*_*ee*_ suppresses pore growth. When particle–epoxy interactions become comparable to epoxy–epoxy interactions (i.e., when approaches 1), the difference in driving forces diminishes, reducing the system’s tendency to reorganize and thereby stabilizing the microstructure. These data show that in order to attain maximum stability in the pore network, it is desirable to have low asymmetry in particle affinity (epoxy vs oil) and also particles that are highly attractive for the epoxy.

**Figure 13:**
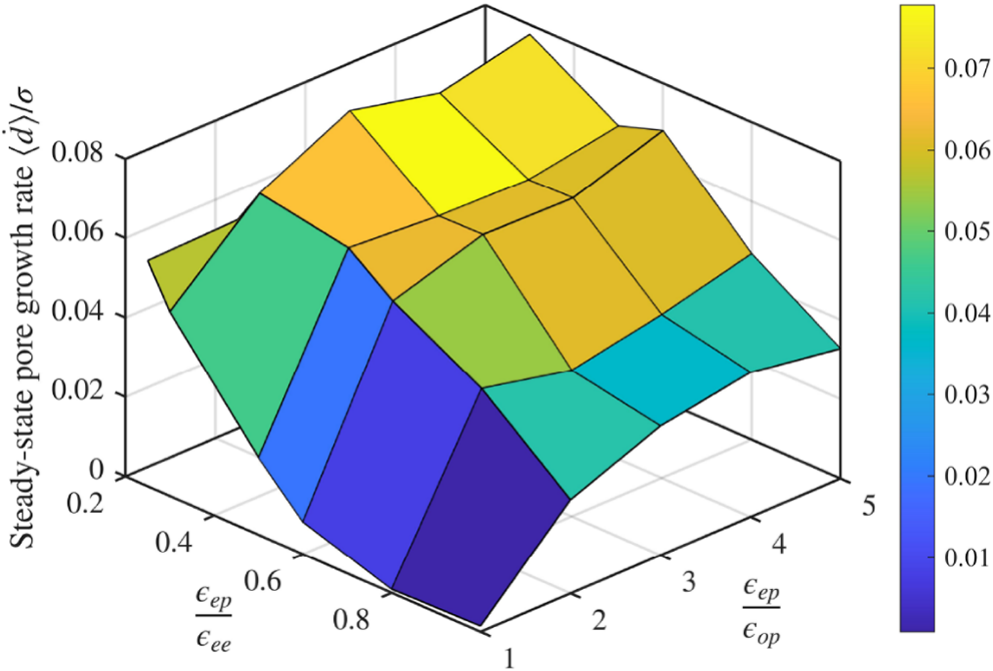
Influence of interaction energy ratios on pore growth rate when *c_s_* = 0.033.

Finally, Figure 14 evaluates how surfactant modifies these trends under different interaction parameter combinations. For *ε*_*ep*_/*ε*_*ee*_ = 1 (red markers), the steady-state pore growth rate is relatively low and shows only a weak dependence on surfactant concentration. In contrast, for *ε*_*ep*_/*ε*_*ee*_ = 0.5 (green markers) and *ε*_*ep*_/*ε*_*ee*_ = 0.25 (blue markers), increasing surfactant concentration generally reduces the steady-state pore growth rate when *ε*_*ep*_/*ε*_*op*_is moderate or large. This reduction is strongest for the *ε*_*ep*_/*ε*_*ee*_ = 0.25 cases. Additional repeated simulations for selected *ε*_*ep*_/*ε*_*ee*_ = 0.25 conditions (*ε*_*ep*_/*ε*_*op*_ = 1, 2, and 5) at surfactant concentrations of 10% and 13.33% showed run-to-run variations of approximately 2–11%, indicating that the small nonmonotonic differences in Figure 14 are within expected statistical uncertainty. Overall, these results indicate that surfactant is most effective at reducing pore growth when particle–epoxy interactions are weak relative to epoxy–epoxy interactions and when the particles preferentially attract epoxy over oil.

**Figure 14:**
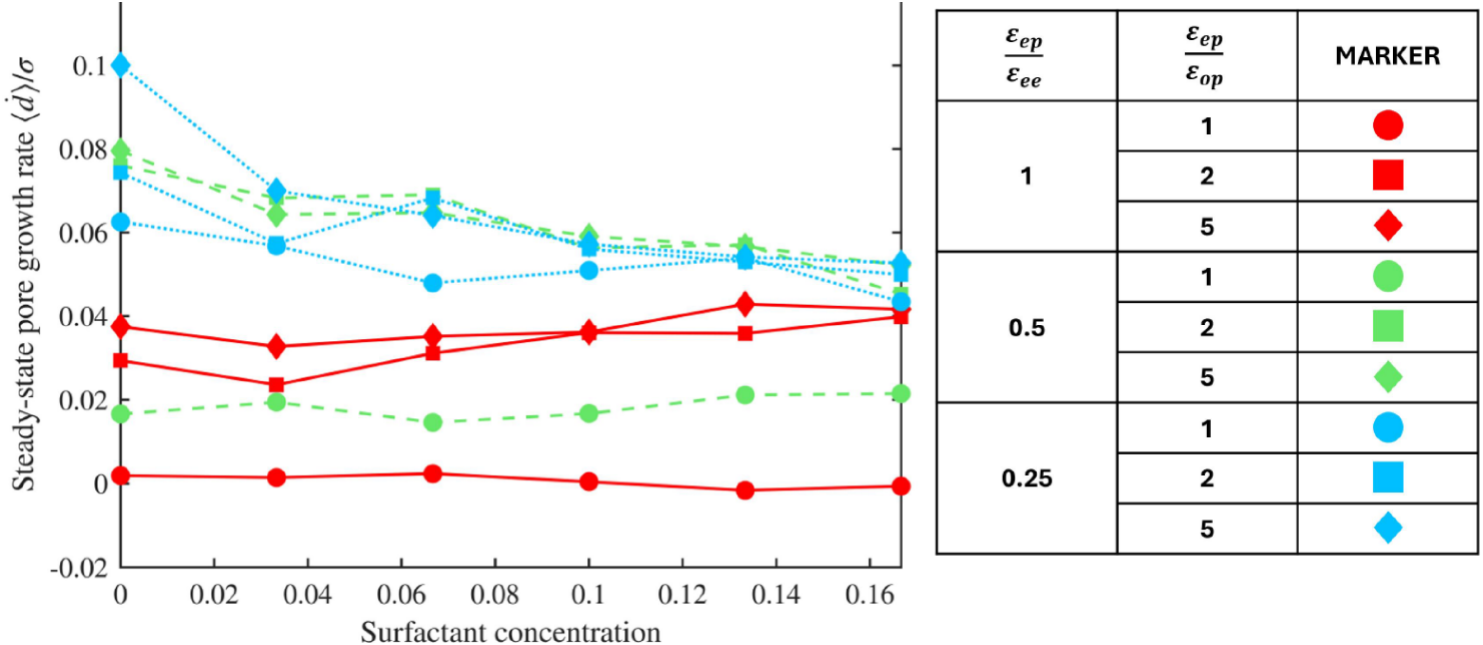
Steady-state pore growth rate as a function of surfactant concentration for different combinations of the interaction energy ratios *ε_ep_*/*ε_ee_* and *ε_ep_*/*ε_op_*.

## Discussion

The results of this study demonstrate that fluid-particle interactions play a central role in determining phase separation dynamics and pore evolution. As shown in Figure 7, when particles are present the temporal scaling of the domain size does not obey traditional scaling exponents. Without particles, we observe exponent α = 1/3 at early times (when diffusion dominates) transitioning toward 2/3 < α < 1 as hydrodynamic interactions/surface tension become more important, as expected based on prior work.^18,38^ However, when particles are present, at early times we observe a much lower exponent of α ≈ 1/6. We hypothesize that the reason for this reduction in the scaling exponent is that particles act as obstacles to diffusion, forcing molecules to take tortuous diffusion paths. Attractive and/or repulsive interactions between particles and molecules are also likely to play a role, but we did not explore sensitivity of the scaling exponent α to interaction energies; we leave this to future work.

Moving on to examine the PSDs we obtained for our simulations; in all cases we observed a transition from monomodal to bimodal PSDs over time. Interestingly, a bimodal PSD was also observed in MINETs with particle sizes less than 100 nm, but not with particle sizes larger than 100 nm.^5^ This gives evidence that our results may be most relevant to small particle MINETs (as expected given that they are obtained from MD simulations). Our results in Figure 11 show that the bimodal character weakens as the particle size increases, albeit to a modest degree over a small range of particle sizes. Nonetheless, this is consistent with experiments.^5^ Interestingly, we also observe bimodal PSDs in systems without particles, indicating that perhaps the bimodality is intrinsic to the phase-separating fluid system, which larger particles disrupt but smaller particles do not.

Using the steady-state pore growth rate ⟨*ḋ*⟩ to quantify MINET stability, our results provide clear trends with interaction energies and composition. First, we note that stability is maximized, in the sense of minimizing ⟨*ḋ*⟩, when all particle interactions are close to neutral, i.e., *ε*_*ep*_/*ε*_*op*_ ≈ *ε*_*ep*_/*ε*_*ee*_ ≈ 1. If particles prefer epoxy over oil (*ε*_*ep*_/*ε*_*op*_ > 1) or epoxy prefers itself over particles (*ε*_*ep*_/*ε*_*ee*_ < 1), the pore structure evolves more rapidly over time. Interestingly, even in the case of neutral particle interactions we find that a MINET structure still forms with particles wetted inside of epoxy domains, as shown in Figure 14. We may have expected that under neutral conditions the particles would be distributed across both oil and epoxy domains, however it seems that the lower volume fraction of epoxy in the system and associated phase separation dynamics still leads to preferential epoxy wetting even though the particles have no thermodynamic preference between oil and epoxy.

Our results also indicate that any change moving away from neutral particle interactions leads to destabilization of the pores (larger growth rate). We find that the steady-state growth rate is slightly more sensitive to *ε*_*ep*_/*ε*_*op*_than it is to *ε*_*ep*_/*ε*_*ee*_. Furthermore, we find that surfactant behaves in essentially the opposite way; surfactant is only effective at stabilizing the pores when particle interactions are not neutral. In other words, the surfactant is only able to reduce the pore growth rate if both *ε*_*ep*_/*ε*_*op*_ > 1 and *ε*_*ep*_/*ε*_*ee*_ < 1. This indicates that a complex interplay between the fluids, particles, and surfactants underlies pore growth. For example, we may expect that surfactant always slows pore growth, since phase separation is always occurring (independent of *ε*_*ep*_/*ε*_*op*_and *ε*_*ep*_/*ε*_*ee*_). However, our results indicate that this is not the case. Finally, Figure 14 suggests a tendency toward similar behavior when *ε*_*ep*_/*ε*_*op*_is large and *ε*_*ep*_/*ε*_*ee*_ is small, as the corresponding ⟨*ḋ*⟩ versus *c*_*s*_ curves become more similar, although they do not collapse onto a single universal curve.

It is worth contrasting our observations here with mechanisms underlying pore evolution in other particle-laden emulsion systems. Bijels form stable networks through jamming of the particles at phase separation interfaces;^2^ we see no evidence of such jamming in our simulations. In capillary suspensions, stability relies on the formation of capillary bridges (e.g., glue) between particles. Particles in our simulations are fully wetted, precluding such a mechanism. PolyHIPEs, which form foam-like porous networks whose stability is governed by pore wall thinning, exhibit similar trends to what is observed here. It has been shown that both surfactant and particles can be used to stabilize pore walls, but the stabilizing effect is most significant when both are used together.^1^ This result is similar to what we observe in Figure 15, despite the fact that no pore wall thinning processes are occurring in our simulations. Furthermore, particles often segregate to phase boundaries in polyHIPEs^1,3^ whereas we saw no evidence of this in our simulations. Nonetheless, this common behavior is worth exploring more fully in future work.

**Figure 15:**
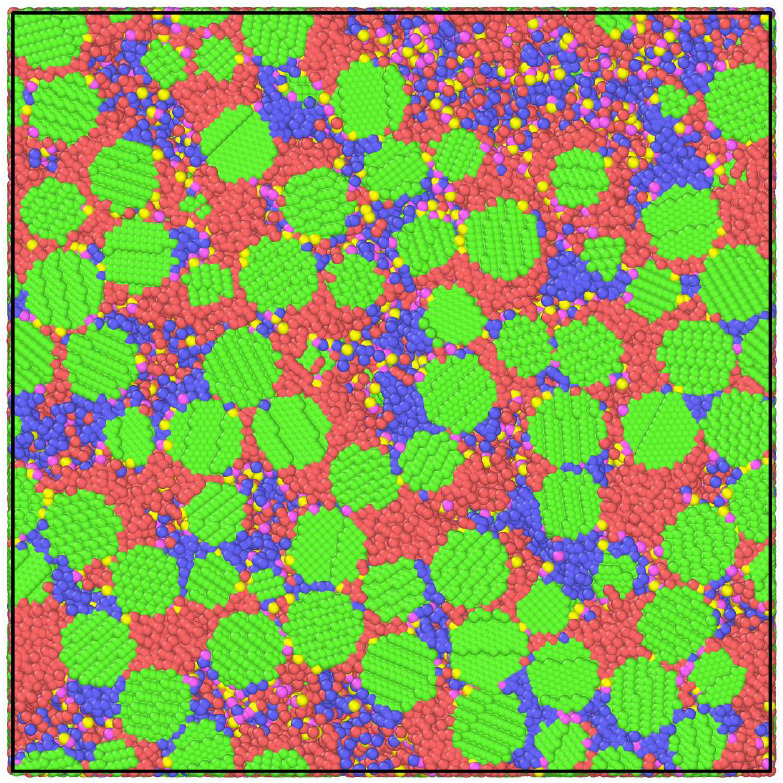
Simulation snapshot from a simulation with neutral particle interactions (*ε_ep_*/*ε_op_* = *ε_ep_*/*ε_ee_* = 1) at nondimensional time t* = 3,000,000. Particles still preferentially wet the (blue) epoxy due to its lower volume fraction, even though they have the equal interaction with the (red) oil.

## Conclusions

We presented a CG-MD study of MINET pore network formation and evolution. We used traditional correlation analyses to quantify the phase separation kinetics from our simulations, obtaining classical behaviors in systems without particles but observing anomalous scaling when particles are introduced. We hypothesized that this anomalous behavior is the result of particles modifying the diffusion kinetics of the fluid species. We then explored the PSD evolution from our simulations, finding in all cases that after a transient period, all simulations transitioned to steady-state pore growth. The steady-state growth rate provides a measure of stability for the MINET system, which can inform MINET processing design. Finally, we explored the sensitivity of the pore growth rate to interaction energies and surfactant concentration, finding that neutral particle interactions and high surfactant concentration led to lower pore growth rates. Future research should build on our relatively simple CG-MD model by incorporating more accurate molecular representations (e.g., polymer chains) and more sophisticated force fields, and also explore the use of continuum-based models such as phase field and lattice Boltzmann. Through these efforts, it may be possible to systematically investigate how particle surface chemical modification, thermal treatment, and external field manipulation influence phase separation kinetics and pore evolution. This will not only provide a deeper understanding of the stabilization mechanisms of MINETs but also provide theoretical guidance for the design of novel porous materials with controllable pore size distribution and enhanced mechanical properties.

## References

[1] Silverstein M. S. (2014). PolyHIPEs:
Recent Advances in Emulsion-Templated Porous Polymers. Prog. Polym. Sci.

[2] Cates M. E., Clegg P. S. (2008). Bijels: A New Class
of Soft Materials. Soft Matter.

[3] Foudazi R. (2021). HIPEs to PolyHIPEs. React. Funct. Polym.

[4] Koos E. (2014). Capillary
Suspensions: Particle Networks Formed through the Capillary Force. Curr. Opin. Colloid Interface Sci.

[5] Hasan M., Patel Y., Gamboa A. R., Grzenda M. J., Saro-Cortes V., Mhatre V., Singer J. P. (2022). Shear-Induced
Macropore-Infused Nanocomposite
Emulsion Thermosets. Adv. Mater. Interfaces.

[6] Stratford K., Adhikari R., Pagonabarraga I., Desplat J.-C., Cates M. E. (2005). Colloidal
Jamming at Interfaces: A Route to Fluid-Bicontinuous Gels. Science.

[7] Mohanty, R. Dynamic Mechanical Properties of Carbon Fiber Reinforced With Macropore-Infused Nanoparticle Emulsion Thermoset (MINETs)Master’s ThesisRutgers University 2023

[8] Patel Y., Sun P. H., Llumiquinga B., Bao N., Shi J., Duran A., Nicholas C., Mohanty R., Cho N., You I. (2026). Enhancing Thermal Conductivity and Flame Resistance
of Carbon Fiber Composites Using CNT-infused Multiphase Graphene Resins. Nanoscale.

[9] Kim J. (2012). Phase-Field
Models for Multi-Component Fluid Flows. Commun.
Comput. Phys.

[10] Krüger, T. ; Kusumaatmaja, H. ; Kuzmin, A. ; Shardt, O. ; Silva, G. ; Viggen, E. M. The Lattice Boltzmann Method: Principles and Practice; Graduate Texts in Physics. Springer International Publishing: Cham, 2017.

[11] Saye, R. I. ; Sethian, J. A. Handbook of Numerical Analysis. Elsevier, 2020, Vol. 21, pp. 509–554.

[12] Meyer M., Mareschal M., Hayoun M. (1988). Computer Modeling of a Liquid–Liquid
Interface. J. Chem. Phys.

[13] Smit B., Hilbers P. A. J., Esselink K., Rupert L. A. M., Van
Os N. M., Schlijper A. G. (1991). Structure of a Water/Oil Interface
in the Presence of Micelles: A Computer Simulation Study. J. Phys. Chem.

[14] Stecki J., Toxvaerd S. (1995). The Liquid–Liquid Interface of Simple Liquids. J. Chem. Phys.

[15] Díaz-Herrera E., Alejandre J., Ramírez-Santiago G., Forstmann F. (1999). Interfacial
Tension Behavior of Binary and Ternary Mixtures of Partially Miscible
Lennard-Jones Fluids: A Molecular Dynamics Simulation. J. Chem. Phys.

[16] Ma W.-J., Maritan A., Banavar J. R., Koplik J. (1992). Dynamics of Phase Separation
of Binary Fluids. Phys. Rev. A.

[17] Keblinski P., Ma W.-J., Maritan A., Koplik J., Banavar J. R. (1993). Molecular
Dynamics of Phase Separation in Narrow Channels. Phys. Rev. E.

[18] Ahmad S., Das S. K., Puri S. (2010). Kinetics of Phase Separation in Fluids:
A Molecular Dynamics Study. Phys. Rev. E.

[19] Ahmad S., Puri S., Das S. K. (2014). Phase Separation
of Fluids in Porous
Media: A Molecular Dynamics Study. Phys. Rev.
E.

[20] Singh A., Puri S. (2015). Phase Separation in
Ternary Fluid Mixtures: A Molecular Dynamics
Study. Soft Matter.

[21] Laradji M., Mouritsen O. G., Toxvaerd S., Zuckermann M. J. (1994). Molecular
Dynamics Simulations of Phase Separation in the Presence of Surfactants. Phys. Rev. E.

[22] Zhang Z., Chakrabarti A. (1994). Phase Separation of Binary Fluids
Confined in a Cylindrical
Pore: A Molecular Dynamics Study. Phys. Rev.
E.

[23] Gelb L., Gubbins K. (1997). Liquid-Liquid Phase Separation in Cylindrical Pores:
Quench Molecular Dynamics and Monte Carlo Simulations. Phys. Rev. E.

[24] Kanda H., Makino H. (2008). Liquid-Liquid Phase Separation of
Binary Lennard-Jones
Fluid in Slit Nanopores. Adsorption.

[25] Li C., Strachan A. (2018). Coarse-Grained Molecular
Dynamics Modeling of Reaction-Induced
Phase Separation. Polymer.

[26] Ferrari E. S., Burton R. C., Davey R. J., Gavezzotti A. (2006). Simulation
of Phase Separation in Alcohol/Water Mixtures Using Two-Body Force
Field and Standard Molecular Dynamics. J. Comput.
Chem.

[27] Paul R., Mitra A., Paul S. (2021). Phase separation
property of a hydrophobic
deep eutectic solvent–water binary mixture: A molecular dynamics
simulation study. J. Chem. Phys.

[28] Chen Q., Zheng J. (2023). Self-Assembly and Structures of Nanoscale Double Emulsion Droplets
through Coarse-Grained Molecular Dynamics Simulations. Soft Matter.

[29] Thompson A. P., Aktulga H. M., Berger R., Bolintineanu D. S., Brown W. M., Crozier P. S., in’t Veld P. J., Kohlmeyer A., Moore S. G., Nguyen T. D., Shan R., Stevens M. J., Tranchida J., Trott C., Plimpton S. J. (2022). LAMMPS
- a Flexible Simulation Tool for Particle-Based Materials Modeling
at the Atomic, Meso, and Continuum Scales. Comput.
Phys. Commun.

[30] Schultz A. J., Kofke D. A. (2018). Comprehensive High-Precision
High-Accuracy Equation
of State and Coexistence Properties for Classical Lennard-Jones Crystals
and Low-Temperature Fluid Phases. J. Chem. Phys.

[31] Potoff J. J., Panagiotopoulos A. Z. (1998). Critical Point and Phase Behavior of the Pure Fluid
and a Lennard-Jones Mixture. J. Chem. Phys.

[32] Caillol J.-M. (1998). Critical
Point of the Lennard-Jones Fluid: A Finite-Size Scaling Study. J. Chem. Phys.

[33] Stukowski A. (2010). Visualization
and Analysis of Atomistic Simulation Data with OVITO–the Open
Visualization Tool. Modell. Simul. Mater. Sci.
Eng.

[34] Giesche H. (2006). Mercury Porosimetry:
A General (Practical) Overview. Part. Part.
Syst. Charact.

[35] Edelsbrunner H., Mücke E. P. (1994). Three-Dimensional Alpha Shapes. ACM Trans. Graph..

[36] Velasco E., Toxvaerd S. (1996). Phase Separation in Two-Dimensional Binary Fluids:
A Molecular Dynamics Study. Phys. Rev. E.

[37] Midya J., Das S. K. (2017). Kinetics of Vapor-Solid
Phase Transitions: Structure,
Growth, and Mechanism. Phys. Rev. Lett.

[38] Siggia E. D. (1979). Late Stages
of Spinodal Decomposition in Binary Mixtures. Phys. Rev. A.

[39] Furukawa H. (1985). Effect of
Inertia on Droplet Growth in a Fluid. Phys.
Rev. A.

[40] Furukawa H. (1987). Turbulent
Growth of Percolated Droplets in Phase-Separating Fluids. Phys. Rev. A.

[41] Bray A. J. (2002). Theory
of Phase-Ordering Kinetics. Adv. Phys.

[42] Huse D. A. (1986). Corrections
to Late-Stage Behavior in Spinodal Decomposition: Lifshitz-Slyozov
Scaling and Monte Carlo Simulations. Phys. Rev.
B.

[43] Majumder S., Das S. K. (2013). Temperature and
Composition Dependence of Kinetics
of Phase Separation in Solid Binary Mixtures. Phys. Chem. Chem. Phys.

